# A Painless and Time-Saving Modified Technique for Simple Renal Cyst Treatment with Single-session Ethanol Sclerotherapy

**DOI:** 10.1038/s41598-020-61842-1

**Published:** 2020-03-19

**Authors:** Hao Zhang, Shi-hua Xiong, Xu-jing Jiang, Lin Li, Ya-yun Zhang, Fa-jin Lyu

**Affiliations:** 1 Department of Radiology, Dianjiang County People’s Hospital of Chongqing, Chongqing, 408300 P.R. China; 2 Department of Urology, Dianjiang County People’s Hospital of Chongqing, Chongqing, 408300 P.R. China; 3 Department of General Surgery, Dianjiang County People’s Hospital of Chongqing, Chongqing, 408300 P.R. China; 4 Department of Pharmacy, Dianjiang County People’s Hospital of Chongqing, Chongqing, 408300 P.R. China; 5 Department of Radiology, First Affiliated Hospital of Chongqing Medical University, Chongqing, 400016, China P.R. China

**Keywords:** Clinical trial design, Urological manifestations

## Abstract

Percutaneous puncture ethanol sclerotherapy is a simple, effective, minimally invasive, and inexpensive procedure to manage symptomatic simple renal cysts. We modified specific technical aspects to balance certain potential intraprocedural factors for ensuring minimal procedural pain and duration as well as maximal clinical therapeutic effects and evaluated the safety and efficacy of this modified technique. A total of 84 eligible patients underwent computed tomography-guided single-session ethanol sclerotherapy using the conventional (group A) or modified (group B) technique. In group B, the puncture route was modified from tansparenchymal to direct for reducing renal injury, and preinjection of low-dose intracystic lidocaine was used to control distending pain caused by ethanol injections; therefore, greater ethanol volumes could be injected for improving the resistance and contact of ethanol with the cyst wall, precluding the need for patient repositioning multiple times to decrease procedural duration. Visual analog scale score for pain and procedural time were significantly higher in group A than in group B. The complication rate was slightly higher in group A than in group B, but the success rate was comparable between the two groups. These results suggest that the modified technique is painless, time-saving, and injury-reducing and can thus improve medical care.

## Introduction

Simple renal cysts (SRCs) are one of the most commonly acquired renal cystic diseases and are frequent in the elderly given that their prevalence increases with age^1^. Symptomatic SRCs present common clinical symptoms such as flank pain or mass, hypertension, and hydronephrosis. Percutaneous ethanol sclerotherapy is a simple, effective, minimally invasive, and inexpensive treatment for symptomatic SRCs^2^. Therefore, it is often the first-line procedure and is widely used in clinical practice; however, the procedure has not been standardized to date^3^. The conventional technique reported by Xu *et al*.^4^ is used in early stages of the disease, and its advantages include the use of three-way drainage tube, which prevents air from entering the cyst, and of high ethanol concentration over repeated cycles of injection–suction, which eliminate the step of ethanol dilution^4^. Moreover, fragments of protein-denatured substances are removed to allow a directly contact between ethanol and epithelial lining of the cyst wall. The computed tomography (CT) value is used to monitor ethanol concentration for improving treatment efficacy^4,5^, which is based on the linear correlation between ethanol concentration/density and CT attenuation; when the CT value is lower than −190 HU, ethanol concentration is considered to be greater than 90%.

However, we have encountered a few problems in the course of our clinical practice. First, this puncture route can damage renal tissues, thus increasing the risk of intracystic hemorrhage and hematuria^6^. Second, patients complain of distending pain caused by ethanol injections^7^. Third, the patient’s position needs to be changed twice to increase the contact area between injected ethanol and cyst wall, which requires more time^4,5^. Pain and prolonged procedural time reduce the patient’s compliance and increase their discomfort. Therefore, these factors should be balanced to ensure minimal procedural pain or time and maximal clinical therapeutic effects.

To this end, we sought to modify specific technical aspects of the conventional procedure, which resulted in positive effects. The modified procedure appeared to be more comfortable, faster, and safer. However, the data have not been fully evaluated. Therefore, this exploratory randomized trial was conducted to evaluate whether our modified sclerotherapy technique for SRCs is superior to the conventional one.

## Materials and Methods

### Ethics

This present study was approved by the ethical committee of Dianjiang County People’s Hospital of Chongqing (Approval number: DJ201603). This was a registered clinical trial (Registration ID: ChiCTR-1900026082; Sep. 20, 2019) performed according to the Declaration of Helsinki. Written informed consent was obtained from all patients prior to participation in this study. All procedures on patients were performed in accordance with the relevant guidelines and regulations (Please refer to Supplementary file 1, 2, 3).

### Study population

Between June 2016 and March 2018, 96 consecutive patients with SRCs who were scheduled to undergo ethanol sclerotherapy were enrolled. All patients enrolled in the study were diagnosed with SRCs (Bosniak class I) using CT examination prior to the procedure and met the following inclusion criteria: cyst diameter between 5–8 cm; presence of symptoms (n = 70) such as flank pain or discomfort, mass, or hypertension; and absence of overt clinical symptom reassurance due to increasing cyst size (n = 26). Patients with peripelvic cysts, previously treated cysts, or procedural contraindications were excluded.

### Treatment methods

Between June 2016 and May 2017, sclerotherapy was performed in 49 patients (49 cysts in group A) using the conventional technique. Between June 2017 and March 2018, after obtaining approval from the institutional review board, sclerotherapy was performed in 47 patients (47 cysts in group B) using the modified technique^8^.

CT-guided single-session 99.9% ethanol sclerotherapy was performed on patients in both groups by the same operator, who has an experience of over 500 interventional procedures. Before the procedure, cyst volume was estimated according to the following formula: V = length × width × height × π/6. The patients were placed in the prone position, and 2% lidocaine was administered for local infiltration anesthesia at the puncture site. A 20-gauge coaxial needle (Monopty 2016B Bard, Tempe, AZ, USA) was used to puncture into SRCs, and a three-way drainage tube was used to connect to the coaxial needle. A sample (20 mL) of cyst fluid obtained during initial aspiration was sent for bacteriological, cytological, and biochemical tests. Patients but the operator was blinded to pre-injecting of intracystic lidocaine to control pain because all the medicinal liquids must be checked by the operator before being injected. During the procedure, patients’ visual analog scale (VAS) score for pain and volumes of each aspiration and injection were recorded separately. Of note, it is necessary to steadily aspirate 1–2 mL of cyst liquid through the syringe without exerting negative pressure to confirm that the needle tip is within the cyst prior injecting fluid (including contrast agent, lidocaine, or ethanol) into the cyst. This procedure also avoids injecting a small amount of gas that may be present in the three-way drainage tube into the cyst.

For group A, the puncture route was designed to pass through a portion of the renal parenchyma, with the tip being located at a distance one-third to half of the cyst diameter. Diluted contrast agent (iohexol, 30% w/v) was then injected to ensure that the cyst was not connected with the collecting system of the kidney and that no contrast material was leaking out. After aspirating most of the cyst fluid, 99.9% ethanol was injected into the cyst at a volume equal to 25% of the volume of aspirated fluid. This aspiration and injection cycle was repeated was with 20 mL ethanol until the aspiration fluid appeared clear on visual inspection. CT was performed to confirm that the CT value was below −190 HU. The patient was placed in the prone and bilateral decubitus positions for approximately 5 min each. All fluid was then aspirated out of the cyst, and the coaxial needle was removed. The patients were escorted back to the ward by a nurse and were discharged the next day if they did not develop complications.

**Figure 1:**
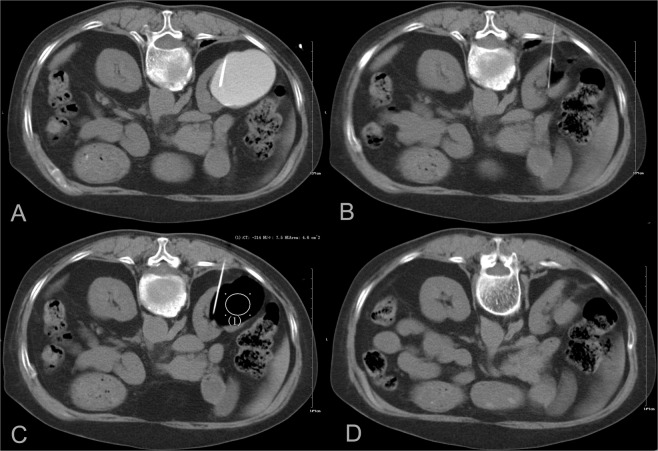
A 74-year-old female with persistent pain in the right loin for 2 months. (**A**) The cyst was not punctured through renal parenchyma considering that the needle tip was located at the cyst base. (**B**) During replacement, at least 10–20 mL of liquid was retained in the cyst for the operator to confirm that the needle tip was inside the cyst. At this point, even though the cyst wall collapses, the needle tip near the cyst base remains within the cyst. (**C**) After completing the second replacement, approximately 75% of the cyst volume of the ethanol was retained in the cyst, with a CT value of −214 HU, indicating an ethanol concentration of >90%, which was retained for 5 min. (**D**) After the procedure, the cyst wall completely collapsed and the cyst cavity disappeared.

**Figure 2:**
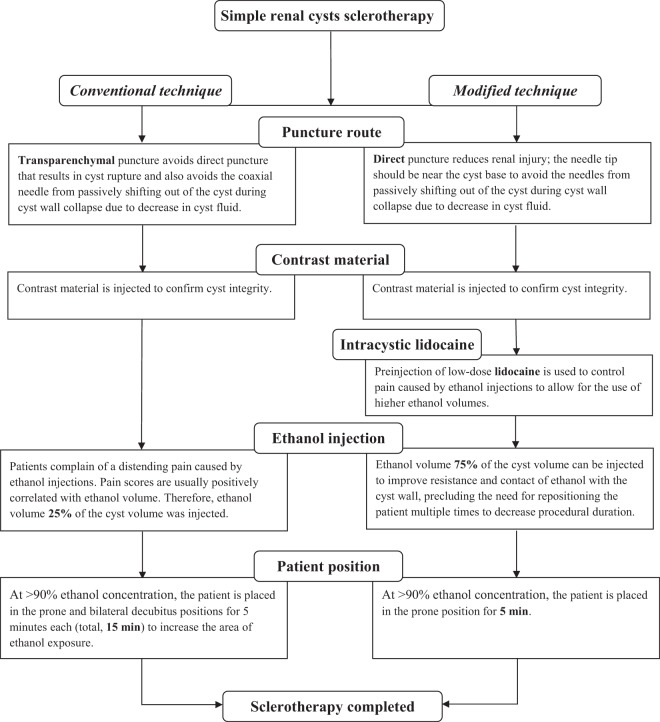
Flow diagram of comparison of the conventional and modified Techniques.

For group B, the puncture route was designed to avoid the renal parenchyma, and the needle tip was located near the cyst base. After injecting diluted contrast agent to confirm normal conditions, 3–5 mL of 2% lidocaine was injected into the cyst and retained for 1 min. Next, based on the estimated cyst volume, most of the fluid within the cyst was aspirated, with retention of only 10–20 mL. This enabled the operator to confirm that the needle tip was within the cyst by steadily aspirating 1–2 mL of fluid. Then, 99.9% ethanol (not more than 200 mL) was injected into the cyst to occupy approximately 75% of the original volume, which was calculated based on the estimated cyst volume. This aspiration and injection cycle with the same volume was repeated. Typically, following completion of the second or third replacement, the ethanol concentration in the cyst cavity exceeded 90% and remained unchanged for 5 min under the same patient position (Figs. 1, 2). Ethanol was then withdrawn, followed by the same procedures performed for patients in group A.

### Data analysis

Pain score for sclerotherapy was determined using a reference VAS, in which 0, 1–3, 4–6, 7–9, and 10 points represented no, mild, moderate, severe, and unbearable pain, respectively. The procedural duration was determined on CT images from the patient’s location scan to the immediate postoperative review. Patients were reexamined by CT at the 12-month follow-up. Cyst size was recorded, and the curative effect was evaluated; over 50–80% reduction in cyst size was considered partial/complete regression.

Normality of distribution of continuous variables was assessed by the Shapiro–Wilk test. Continuous variables with normal distribution were reported as mean and standard deviation; the means of variables between the two groups were compared by independent sample Student’s test. Continuous variables with non-normal distribution were presented as median (interquartile range), and the means of variables between the two groups were compared by the Mann–Whitney U test. Pain score and efficacy were classified as ordered categorical variables and analyzed using the Mann–Whitney U test between groups. For other categorical variables, frequencies were compared using Pearson χ^2^ or Fisher’s exact test, as appropriate. All data were analyzed using SPSS version 22.0 for Windows (SPSS Inc., Chicago, IL, USA). A value of *p* < 0.05 was considered statistically significant.

## Results

**Figure 3:**
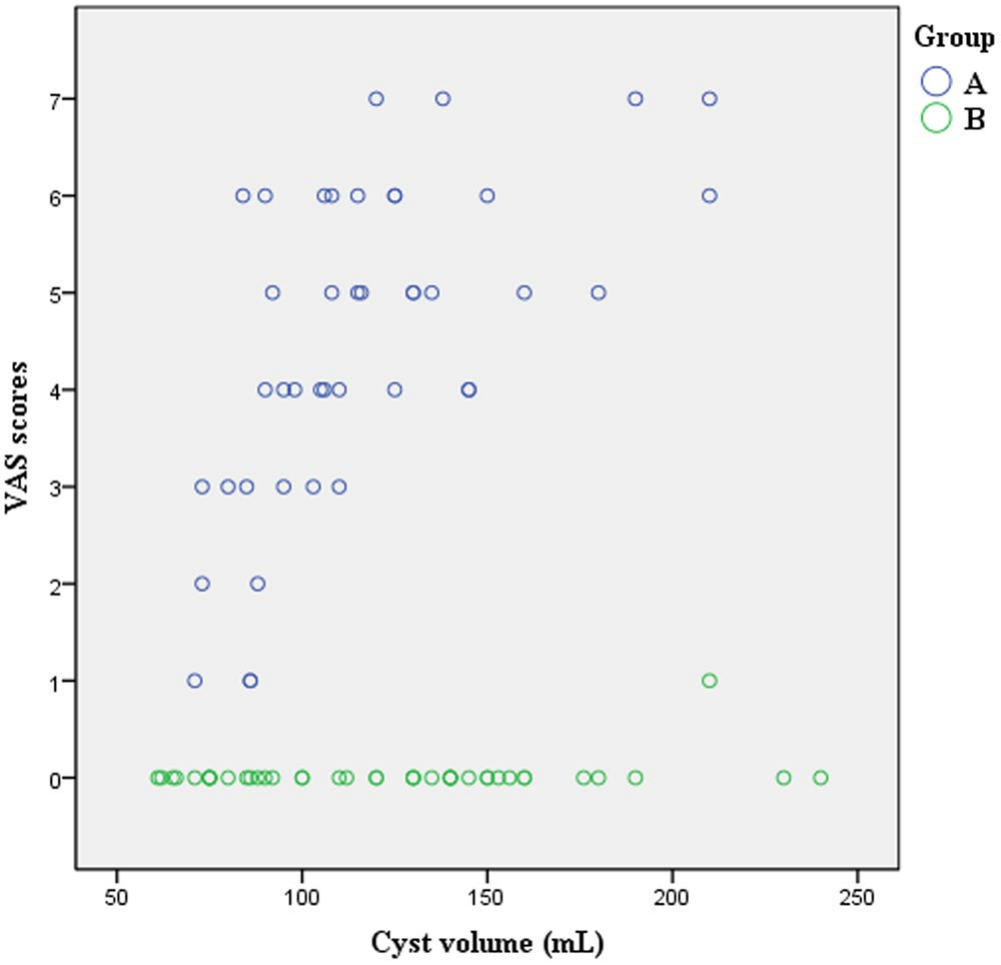
Scatter plot of VAS score. VAS score in group A (mean, 3.6 ± 1.5; range, 1–7) was positively correlated with ethanol volume injected (*r* = 0.992, *p* < 0.05).

**Figure 4:**
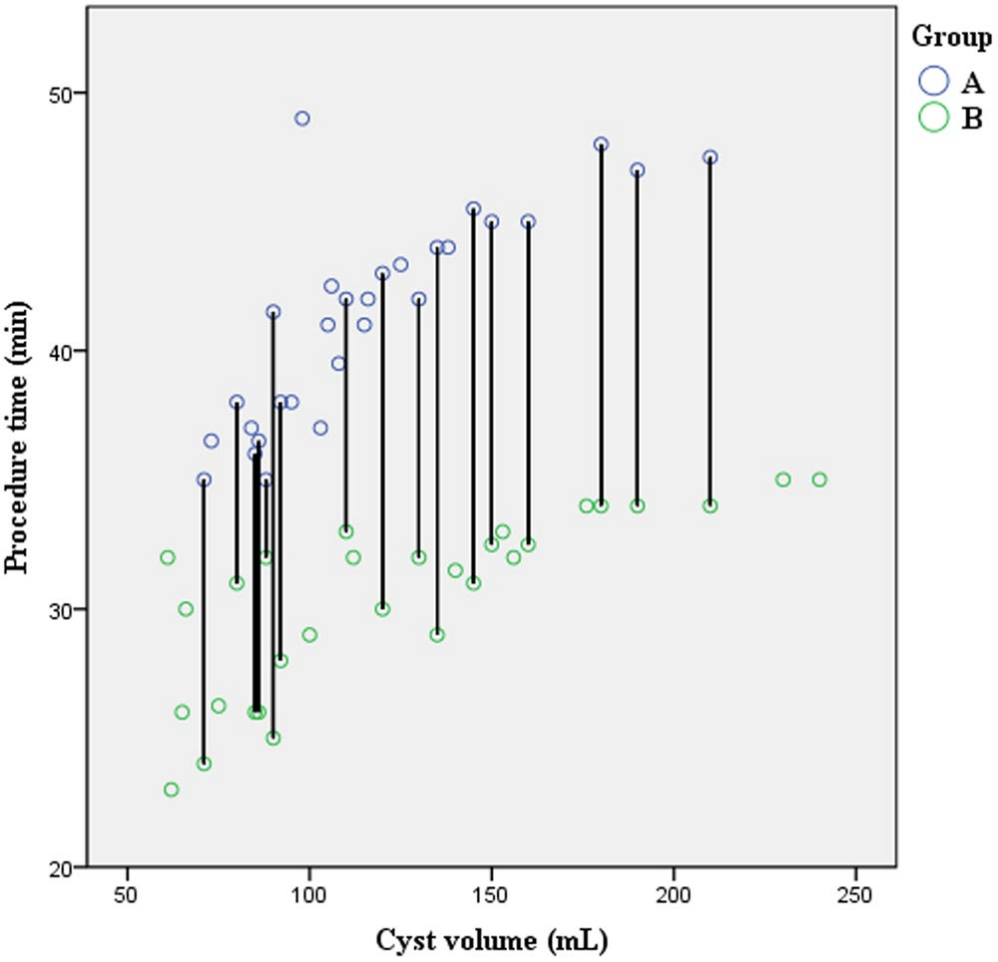
Scatter plot of procedural time. Average difference between groups A (41.4 ± 3.8) and B (30.3 ± 3.4) was approximately 11 min.

**Figure 5:**
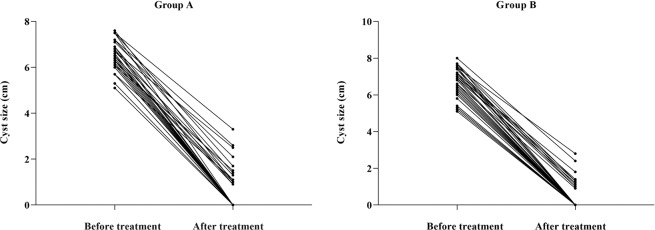
Cyst size before and after treatment in groups (**A,B**). Mean cyst size ± standard deviation (range) before and after treatment was 6.5 ± 0.6 (5.0–7.6) and 0.5 ± 0.9 (0–3.3) cm (*p* < 0.01) in group A and 6.6 ± 0.8 (5.1–8.0) and 0.4 ± 0.7 (0–2.8) cm (*p* < 0.01) in group (**B**).

Twelve patients were lost to follow-up. Finally, a total of 84 eligible patients with SRCs (n = 84) were enrolled in the study. Demographic and SRCs characteristics of the patients are summarized in Table 1. Upon biochemical examination, no abnormality was found in cyst fluid. Relevant treatment parameters are shown in Table 2. Median VAS (Fig. 3) in group A were significantly higher (*p* < 0.01) than that in group B. Patients in group A complained of different degrees of pain, whereas only one patient in group B reported mild swelling but no pain. In this case, the cyst had a volume of 210 mL and was in the early stages of our modified technique experience. Only 3 mL lidocaine was injected into that cyst, which might have been inadequate. In one case in group A, acute intracystic hemorrhage occurred following the puncture of an 88-mL cyst. One unit of hemocoagulase was injected into the cyst and retained for 5 min. Then, it was repeatedly replaced with normal saline until the liquid showed no obvious blood color. Procedural time for that case was prolonged to 49 min. Microscopic hematuria occurred in two patients in group A. Ethanol exposure and procedural times were higher in group A than in group B (Fig. 4). Symptoms of flank pain or mass were relieved, and hypertension was partially relieved. The 12-month follow-up showed similar success rates (partial and complete regression) between the two groups (Fig. 5).

**Table 1:** Demographic and renal cyst characteristics.

Characteristics		Group A (*n* = 42)	Group B (*n* = 42)	*P* value
Age (years)		65 (60–71)	65 (58–72)	0.707
Sex	Male/Female	24 (57.1)/18 (42.9)	26 (61.9)/16 (38.1)	0.657
Laterality	Left/Right	22 (52.4)/20 (47.6)	18 (42.9)/24 (57.1)	0.382
Location	Upper pole/Lower pole	18 (42.9)/14(33.3)	15 (35.7)/14 (33.3)	0.717
	Middle portion	10 (23.8)	13 (31.0)	
Orientation	Anterior/Posterior	14 (33.3)/4 (9.5)	8 (19.0)/6 (14.3)	0.338
	Medial/Lateral	4 (9.5)/20 (47.6)	8 (19.0)/20 (47.6)	
Clinical Presentation				0.643
Flank pain		24 (57.1)	19 (45.2)	
Flank mass		4 (9.5)	3 (7.1)	
Hypertension		3 (7.1)	5 (11.9)	
Anxiety		11 (26.2)	15 (35.7)	
Estimated cyst volume (mL)		105 (85–127)	127 (86–158)	0.161
Actual cyst volume (mL)		109 (90–131)	125 (84–151)	0.567

Note—Values are presented as median (interquartile range) or number (percentage).

**Table 2:** Relevant treatment parameters.

Parameter	Group A (*n* = 42)	Group B (*n* = 42)	*P* value
Puncture depth (cm)	7.3 ± 1.2	7.2 ± 1.2	0.525
Puncture angle (°)	8 (4–30)	11 (4–27)	0.830
Ethanol exposure time (min)	19.1 ± 2.9	9.6 ± 1.7	<0.001
Procedure time (min)	41.4 ± 3.8	30.3 ± 3.4	<0.001
VAS	3 (3–5)	0 (0–0)	<0.001
Complication	3 (7.1)	0 (0)	0.241
Microscopic hematuria	2	0	
Intracavitary hemorrhage	1	0	
Clinical symptomatic remission	40 (95.2)	38 (90.0)	0.676
Flank pain	24	19	
Flank mass	4	3	
Hypertension	1	1	
Anxiety	11	15	
Therapeutic evaluation			0.695
Complete regression rate	38 (90.5)	39 (92.9)	
Partial regression rate	4 (9.5)	3 (7.1)	

Note—Values are presented as mean ± standard deviation, median (interquartile range), or number (percentage).

## Discussion

SRCs are usually asymptomatic; however, flank pain is a relatively common symptom in individuals with symptomatic SRCs. It possibly occurs due to dilation of the renal capsule; furthermore, some symptoms may appear secondary to obstruction of the collecting system^9^. Bryniarski *et al*.^10^ have reported that the prevalence of hypertension in patients with SRCs was 43.3% (91/210 patients), of which 61.7% (56/91 patients) were relieved of hypertension following cyst sclerotherapy or laparoscopic. Therefore, after the cyst was ablated, the associated clinical symptoms were relieved; residual cysts without symptoms do not require further intervention^7,11^. Furthermore, some patients without overt clinical symptoms opt to undergo treatment because their anxiety increases with increasing cyst volume^5,11–13^.

Presence of a cyst with a diameter of >4 cm is one of the inclusion criteria for SRC sclerotherapy^14,15^. Large cysts (>8 cm) tend to fold after cavitary collapse. Sometimes, there is no contact between injected ethanol and cyst, and such contact, if any, is less effective; therefore, laparoscopy is the preferred option in such cases^2,3,14^. Residual cysts detected during early follow-up after sclerotherapy may be secondary to transient, reactive, or inflammatory fluid accumulation, which may take 6–12 months to completely resolve^16^. Therefore, cyst diameter in this comparative study was controlled at 5–8 cm, with a follow-up time of 12 months. Although peripelvic cyst is not a contraindication for sclerotherapy^12,17^, ethanol leakage at the ureteropelvic junction may lead to fibrosis and advanced stenosis^18,19^. Therefore, for the sake of safety, peripelvic cysts were excluded from this study.

Ultrasound and CT are commonly used as a guidance modality. Ultrasound has the advantages of being cost-effective, real-time, and non-radiative, although it is much more dependent upon operator skills than CT^13^. CT is preferable in obese patients or when the cyst is unclearly visualized and a safe access route cannot be identified.

Transparenchymal and direct puncture are both acceptable methods in the literature and are usually left to the discretion of operator, primarily dictated by the cyst location^9,17^. However, some operators still prefer to use the former, as shown in previous studies^4,5,20^, because it avoids direct puncture leading to cyst rupture, and the coaxial needle is passively shifted out of the cyst during cyst wall collapses as the fluid decreases. However, this path may damage renal parenchyma. In a study by Dell’atti *et al*.^6^, incidence of hematuria and intracapsular hemorrhage was 8.3% (3/36 cases) and 2.7% (1/36 cases), respectively. Data on complications obtained in the present study were insufficient to determine whether they were caused by transparenchymal puncture. Nonetheless, direct puncture avoids renal parenchymal injuries; thus, we recommend it for suitable cases. This notion is consistent with the view put forth by Lee *et al*. that a cyst should be punctured as atraumatically as possible to decrease bleeding into it^9^. In our experience, care should be exercised to control patient’s breathing and to minimize the risk of cyst rupture during direct puncture. During the procedure, as the fluid in the cyst decreases, the wall collapses toward the cyst base. Therefore, the needle tip should be near the cyst base.

The most common complication of ethanol sclerotherapy is distending lumboabdominal pain^3,6,7,15^, which results in a higher VAS score. Yonguc T *et al*. reported that mean VAS scores of the overall and severe case were 4.26 ± 1.99 and 5.1 ± 2.1, respectively^15^. Moreover, analgesics may be applied^6^ in some cases, while the operator is forced to inject smaller ethanol volumes in others^21^. In severe cases, the procedure is often suspended or discontinued^7,12,15,21^. Although studies have recommended the use of ethanol, 20% hypertonic saline can also be used to avoid pain^20^. Lidocaine is a commonly used and readily available local anesthetic that is inexpensive, blocks painful stimuli, and works rapidly. Because lidocaine controls pain^7,22^, greater ethanol volume can be injected, improving the contact of ethanol with the cyst wall. This prevents the need for repositioning the patient multiple times, leading to decreased procedural duration.

In previous reports on single-session sclerotherapy, 95–99.9% ethanol at a volume of 15–50% of the cyst volume was used as a sclerosing agent^3,7^ (maximum injected ethanol volume of 200 mL)^5,23^ without adverse effects^23^. The exposure time was 5–40 min, and most cases achieved good results^3,24^. Use of high ethanol concentrations can lead to dehydration, protein degeneration, and necrosis of epithelial cells of the monolayer cyst lining within 1–3 min^25^. Additionally, ethanol induces aseptic inflammation of the cyst wall, and after the cystic fluid is exhausted, the wall collapses and the adhesion remains closed^5^. Ethanol gradually penetrates the fibrous capsule in approximately 4–12 h, leading to minimal local or systemic adverse reactions^25^. The outcome of the volume of ethanol (>90% V/V) and time of exposure to it were nearly the same groups A and B; however, the dose–time effect value was lower than the value reported in most previous sclerotherapy treatments^3,24^. Therefore, systemic reactions, which are usually caused by large or prolonged exposure^7,9^ to ethanol, did not occur in either study group. Moreover, no major complications occurred, perhaps because of the small sample size, meticulous procedure, and inherent safety of the procedure^26^.

The modified technique is painless, requires a short time, and minimizes renal parenchymal injury, thereby ensuring a rapid and smooth procedure. Likewise, it is also suitable for ultrasound-guided sclerotherapy, wherein only the guidance technique is different. Moreover, if certain operators are accustomed to transparenchymal puncture or in case the puncture path cannot avoid the renal parenchyma, injection of intracystic lidocaine and subsequent procedures can still be performed with reference to the modified technique to produce the same effect. Acquiring details of patient improvements enables to minimize renal parenchymal injury, pain, anxiety, and discomfort caused by prolonged procedures, thus achieving better medical care.

Our research has several limitations that warrant further discussion. First, it is not a prospective randomized controlled trial, and there may be a selection bias. Second, the sample size of patients treated with the modified technique is small; thus, further randomized studies in a large patient cohort are warranted to confirm our results. Finally, ethanol sclerotherapy was performed even in asymptomatic patients with SRCs.

In summary, we recommend direct puncture of the cyst to avoid renal parenchymal injury, preinjection of intracystic lidocaine to control pain, and increase in ethanol volume/exposure area without changing the patient position to shorten the procedural duration. Furthermore, the modified technique is safe and effective and can thus improve medical care.

## Supplementary information

Supplementary information

Supplementary information2 

Supplementary information3 

## Data Availability

The data used to support the findings of this study are included within the article.
