# Supplementary figures and images for: A Painless and Time-Saving Modified Technique for Simple Renal Cyst Treatment with Single-session Ethanol Sclerotherapy
Source: Sci Rep. 2020 Mar 19;10:5019. doi: 10.1038/s41598-020-61842-1 (PMC7081224; doi:10.1038/s41598-020-61842-1)

## CONSORT 2010 Flow Diagram

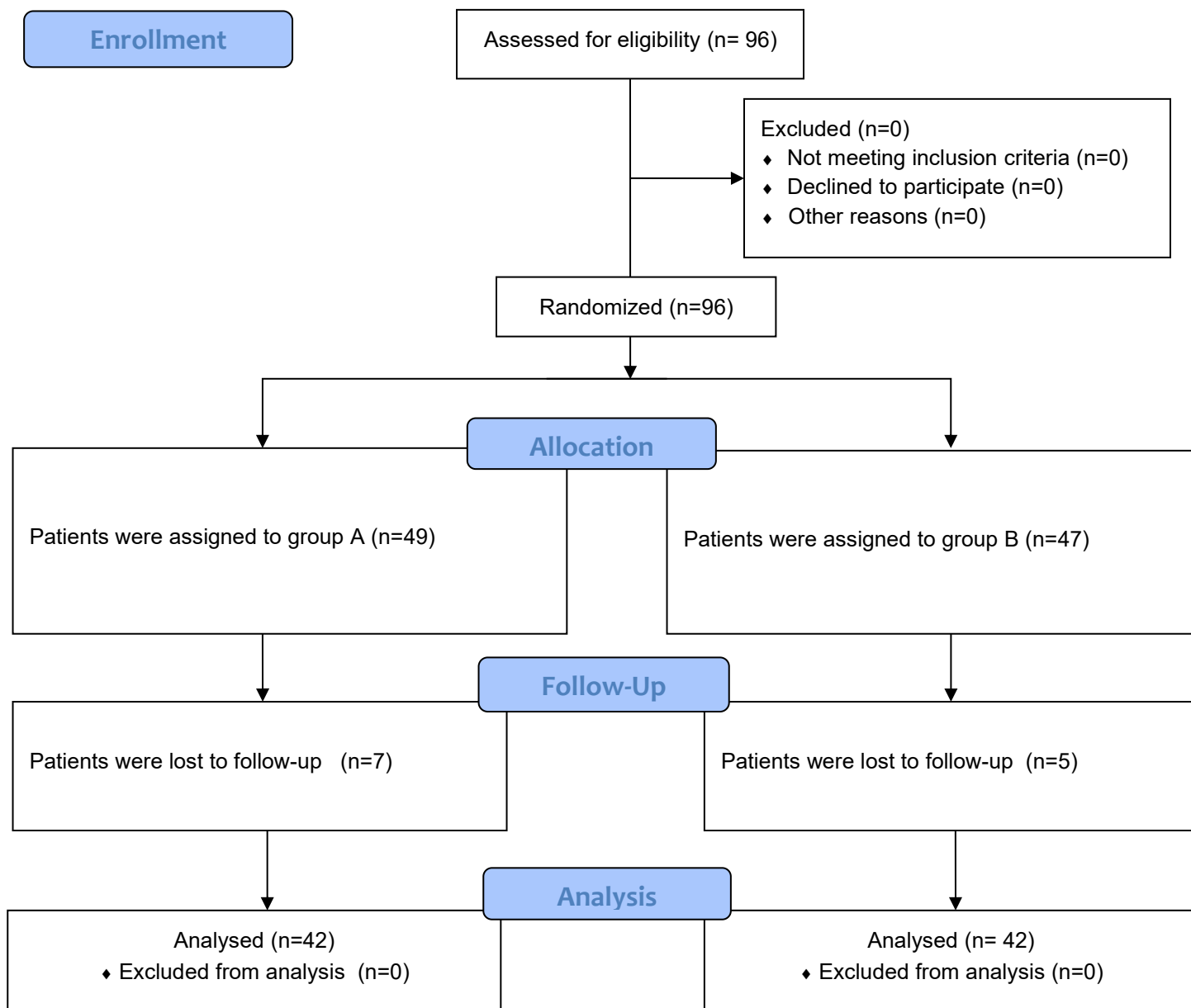

Supplement: Supplementary file 2 — Supplementary information2 [file 41598_2020_61842_MOESM2_ESM.pdf]
