# Supplementary material for: A Painless and Time-Saving Modified Technique for Simple Renal Cyst Treatment with Single-session Ethanol Sclerotherapy
Source: Sci Rep. 2020 Mar 19;10:5019. doi: 10.1038/s41598-020-61842-1 (PMC7081224; doi:10.1038/s41598-020-61842-1)
Supplement: Supplementary file 3 — Supplementary information3 [file 41598_2020_61842_MOESM3_ESM.pdf]

Comparison of Conventional versus Modified Techniques for Simple Renal Cysts Treatment with Single-session Ethanol Sclerotherapy

Edition：1.0
Original Protocol: March 30 2016

Principle Investigator: Zhang Hao
Research Site: Dianjiang County People's Hospital of Chongqing, China
Address: 116 BeiWai St., GuiXi Town, DianJiang County, Chongqing, China, 408300
Phone: +86 023 8565 3623

Project summary

Subjects	Simple Renal Cysts Treatment with Single-session Ethanol Sclerotherapy
Objects	To investigate the safety and efficacy of modified technique ethanol sclerotherapy for simple renal cysts
Sample Size	84 qualified subjects
Study Duration	June 2016- March 2018
Patient screening criteria	**Inclusion criteria：**<br>1. Age: ⩾ 18 years old, male or female;<br>2. Cyst diameter between 5-8 cm with symptoms;<br>3. Absence of overt clinical symptom reassurance due to increasing cyst size;<br>**Exclusion criteria：**<br>Patients with peripelvic cysts, previously treated cysts, or procedural contraindications
Study Design	Subjects were randomly divided into two groups:<br>1) control group, sclerotherapy was performed in 49 patients (49 cysts in group A) using the conventional technique.control group.<br>2) case group, sclerotherapy was performed in 47 patients (47 cysts in group B) using the modified technique.
Main efficacy index	Visual analog scale
Secondary efficacy index	Procedure time
Main safety indicator	Complication rate

1. Background

Simple renal cysts (SRCs) are one of the most commonly acquired renal cystic disease and are commonly found in the elderly, as the prevalence rate increases with age [1]. It causes common clinical symptoms including flank pain or mass, hypertension, and hydronephrosis. Percutaneous ethanol sclerotherapy is the simple, effective, minimally invasive, and inexpensive treatment for symptomatic SRCs [2]. Therefore, it is often the first-line procedure and is widely used in clinical practice; however, it has not been standardized to date [3]. The conventional technique reported by Xu et al. [4] was used in the early stages. It has advantages such that the three-way drainage tube prevents air from entering the cyst and the ethanol concentration is increased by repeated cycles of injection-suction, thereby eliminating the dilution of ethanol [4]. Fragments of protein-denatured substances are also removed to make the ethanol to directly contact the epithelial lining of the cyst wall. The CT value is used to monitor the ethanol concentration for improving the efficacy of treatment [4, 5], which is based on the linear correlation between ethanol concentration/density and CT attenuations; when the CT value is lower than −190 HU, the ethanol concentration is considered to be greater than 90%.

However, we have encountered a few problems in the course of our clinical practice. First, this puncture path can damage kidney tissue, increasing the risk of intracystic hemorrhage and hematuria [6]. Second, patients complain of a burning sensation or distending pain caused by ethanol injections [7]. Third, the patient's position needs to be changed twice to increase the contact area between the injected ethanol and cyst wall, which requires more time [4, 5]. Pain and longer procedure time reduce the patient's coordination and increase discomfort. These factors must be balanced to ensure minimal procedural pain or time and maximal clinical effect of the treatment.

Subsequently, we tried to modify some of the conventional techniques, and these modifications resulted in positive effects. The procedure appeared to be more comfortable, faster, and safer. However, these data have not been fully evaluated. Therefore, this exploratory randomized trial was conducted to evaluate whether modified sclerotherapy for SRCs is superior to conventional technique.

References:

1. Choi JD. Clinical characteristics and long-term observation of simple renal cysts in a healthy Korean population. Int Urol Nephrol 2015;48:319–324.
2. Efesoy O, Tek M, Bozlu M, et al. Comparison of single-session aspiration and ethanol sclerotherapy with laparoscopic de-roofing in the management of symptomatic simple renal cysts. Turk J Urol, 2015, 41:14–19.

3. Eissa A, Sherbiny AE, Martorana E, et al. Non-conservative management of simple Renal cysts in adults: A comprehensive review of literature. Minerva Urol Nefrol 2018;70:179–192
4. Xu XX, Du Y, Yang HF, Zhang Q, Li Y, Zee CS. CT-guided sclerotherapy with ethanol concentration monitoring for treatment of renal cysts. AJR Am J Roentgenol 2011;196:W78–W82.
5. Yu JH, Du Y, Li Y, Yang HF, Xu XX, Zheng HJ. CT-guided sclerotherapy for simple renal cysts: value of ethanol concentration monitoring. Korean J Radiol 2014;15:80–86.
6. Dell'Atti L. Comparison between the use of 99% ethanol and 3% polidocanol in percutaneous echoguided sclerotherapy treatment of simple renal cysts. Urol Ann 2015;7:310–314.
7. Zerem E, Imamovíc G, Omerovíc S. Symptomatic Simple Renal Cyst: Comparison of Continuous Negative-Pressure Catheter Drainage and Single-Session Alcohol Sclerotherapy. AJR Am J Roentgenol 2008;190:1193–1197.

2. Study goals and objectives

2.1 Research purposes
To investigate the safety and efficacy of modified technique ethanol sclerotherapy for simple renal cysts.

2.2 Main study endpoint
Visual analog scale of intraprocedural pain (0-10): Pain score for sclerotherapy was determined using a reference visual analog scale, in which 0, 1–3, 4–6, 7–9, and 10 points represented no pain, mild pain, moderate pain, severe pain, and unbearable pain, respectively.

2.3 Secondary study endpoint
Procedure time: The procedural duration was determined by CT images from the patient's location scan to the immediate postoperative review.

3. Study Design

This is a randomized study evaluating the safety and effectiveness of modified technique ethanol sclerotherapy for simple renal cysts.

3.1 Study Population
The Investigator enrolled subjects expected to meet the study entry criteria to participate in the study.

Eligible subjects will be enrolled and randomized divided into two groups: case and control groups. CT follow-up was performed after 12-month.

3.2 Subject Selection

3.2.1 Inclusion criteria：
1) Age: ⩾ 18 years old, male or female;
2) Cyst diameter between 5-8 cm with symptoms;
3) Absence of overt clinical symptom reassurance due to increasing cyst size;

3.2.2 Exclusion criteria：
Patients with peripelvic cysts, previously treated cysts, or procedural contraindications

3.2.3 Termination criteria
1) The subject withdraws the informed consent and requests to withdraw;
2) The investigator believes that it is necessary to withdraw from the study.

3.2.4 Withdraw criteria
1) Failure to complete clinical trial studies, failure to evaluate safety and efficacy;

2) Serious violation of this study protocol, not according to the method of treatment.

4. Study Methods

4.1 Treatment methods

CT-guided single-session 99.9% ethanol sclerotherapy was performed on patients from both the groups by the same operator who has more than 500 interventional procedures experience. Before the procedure, the cyst volume was estimated according to the formula V = length × width × height × /6.

For group A, the puncture path is designed to pass through a portion of the renal parenchyma, with the tip being located at 1/3 to 1/2 of the cyst diameter. Diluted contrast material (iohexol, 30% w/v) is then injected to ensure that the cyst was not connected with the collecting system of the kidney and no contrast material was leaking out. After most of the cyst fluid had been aspirated, 99.9% ethanol was injected into the cyst, with a volume equal to 25% of the volume of the aspirated fluid. This aspiration and injection cycle with 20 mL was repeated, until the aspiration fluid appeared clear by visual inspection. CT was performed to confirm that the CT value was below −190 HU. The patient was placed in the prone and bilateral decubitus positions for approximately 5 minutes in each position. All fluid was then aspirated out of the cyst and the coaxial needle was removed. The patient was escorted back to the ward by a nurse and was discharged the next day if they did not develop complications.

For group B, the puncture path was designed to avoid the renal parenchyma, and thus the needle tip was located near the cyst base. After diluted contrast agent was injected to confirm no abnormal condition, 3 – 5 mL of 2% lidocaine was injected into the cyst and retained for 1 min. Next, based on the estimated cyst volume, most of the fluid within the cyst was aspirated, with a retention of only 10 – 20 mL. This enabled the operator to confirm that the needle tip was within the cyst by steadily aspirating 1 – 2 mL of fluid. Then, 99.9% ethanol (not more than 200 mL) was injected into the cyst to occupy approximately 75% of the original volume, which was calculated based on the estimated cyst volume. This aspiration and injection cycle with the same volume was repeated. Generally, following completion of the second or third replacement, the ethanol concentration in the cyst cavity exceeds 90% and remains unchanged for 5 min under the same patient position. The ethanol was then withdrawn, followed by performing the same procedures performed in patients from group A.

4.2 Data analysis

Pain score for sclerotherapy was determined using a reference VAS, in which 0, 1 – 3, 4 – 6, 7 – 9, and 10 points represented no pain, mild pain, moderate pain, severe pain, and unbearable pain, respectively. The procedural duration was determined by CT images from the patient ’ s location scan to the immediate postoperative review. Patients were reexamined by CT examination at the 12-month follow-up. Size of cysts

was recorded, and the curative effect was evaluated; over 50% – 80% reduction in cyst size was considered partial/complete regression..

The normality of distribution of continuous variables was assessed by the Shapiro–Wilk test. Continuous variables with normal distribution were reported as mean ± standard deviation; the mean values of the two groups of variables were analyzed by independent sample Student's test. Continuous variables with non–normal are presented as median (interquartile range), and the mean values of the two groups of variables were compared by the Mann–Whitney U test. Pain score and efficacy evaluation were classified as ordered categorical variables and assessed using the Mann–Whitney U test between groups. For other categorical variables frequencies were compared using Pearson $\chi 2$ or Fisher's exact test when appropriate. All data were analyzed by SPSS version 22.0 for Windows (SPSS Inc., Chicago, IL, USA). A value of $p < 0.05$ was considered statistically significant.

5. Effectiveness

5.1 Main endpoints

The pain score of sclerotherapy for simple renal cysts.

5.2 Secondary endpoints

The operation time of sclerotherapy for simple renal cysts.

6. Safety Consideration

6.1 Adverse event (AE)

An adverse event means any untoward medical occurrence associated with the use of a treatment in humans, whether or not considered treatment related. An adverse event (also referred to as an adverse experience) can be any unfavorable and unintended sign, symptom, or disease temporally associated with the use of a treatment, without any judgment about causality.

According to regulations, events occurring before and after treatment are also considered to be AEs. Therefore, the safety monitoring AE or SAE report should begin with the subject entering the trial (signing informed consent) until the end of the trial visit.

6.2 AE classification

AEs are classified as 0-5 (NCI-CTCAE 4.0) according to NCI's Common Acute and Subacute Toxicity Grading Criteria. AEs not listed in the NCI Toxicity Grading Criteria can be judged according to the following criteria:
I degree (slight): has an uncomfortable feeling, but does not affect normal daily activities;
II degree (moderate): The degree of discomfort is sufficient to reduce or affect normal

daily activities;
III degree (severe): a daily activity that cannot work or is normal;
IV degree (fatal): disabling or lethal.

6.3 Collection of AE

Details of all AEs occurring during the study must be recorded on the AE form with the following information:

- Description of the event
- Dates of onset and resolution
- Severity
- Action taken
- Outcome
- Relationship to investigational product
- Whether the AE is serious or not

All AEs will be documented in the subject's source documents (e.g. medical records) and eCRF. Abnormal laboratory test data will be recorded on the CRF form, followed up until normal or at the end of the study.

6.4 Serious Adverse Event

6.4.1 A ***serious adverse event*** (SAE) or suspected adverse reaction is any untoward medical occurrence that, in the view of either the investigator or sponsor, it:

- Results in death;
- Is considered to be life-threatening;
- Requires inpatient hospitalization or prolongation of existing hospitalization;
- Results in persistent or significant disability, incapacity or substantial disruption of the ability to conduct normal life functions;
- Results in a congenital anomaly or birth defect;
- Is an important medical event that may not result in death, be life-threatening, or require hospitalization but may be considered serious when, based upon appropriate medical judgment, they may jeopardize the patient or subject and may require medical or surgical intervention to prevent one of the outcomes listed in this definition.

6.4.2 Hospitalization

Adverse events in clinical studies that result in hospitalization or prolonged hospital stay should be considered serious adverse events. Any initial admission to a medical facility (even if it is shorter than 24 hours) is in compliance with this standard.
Hospitalization does not include the following:

- Rehabilitation institution
- Nursing home
- Conventional emergency room admission
- On-the-spot surgery (eg outpatient/day/apro-bed surgery)
- Hospitalization or lengthening of hospital stays that are not associated with

worsening adverse events are not serious adverse events in themselves, such as:

- Due to the admission of the original disease, there are no new adverse events, and there is no aggravation of the original disease (such as: in order to check the laboratory abnormalities that persisted until the test);
- Hospitalization for management reasons (eg annual routine medical examination);
- Hospitalization as specified in the trial protocol during clinical trials (eg, as required by the protocol);
- Elective hospitalization (such as elective cosmetic surgery) that is unrelated to the deterioration of adverse events;
- The scheduled treatment or surgery should be recorded throughout the trial protocol and/or the individual's individual baseline data;
- Admitted to hospital for blood use only.

Diagnostic or therapeutic invasive (eg surgery), non-invasive procedures should not be reported as adverse events. However, if the disease status leading to this operation meets the definition of adverse events, it should be reported. If the acute appendicitis that occurs during the adverse event reporting period should be reported as an adverse event, the appendectomy should be recorded as the treatment of the adverse event.

6.4.3 SAE reporting procedure

Reports of serious adverse events should begin with the signing of the informed consent form from the subject until the study end. During the trial, if serious adverse events occur, they must be reported to the clinical monitor and the main investigator within 24 hours. At the same time, fill out the "Clinical Research Serious Adverse Event (SAE) Report Form", signed and dated, and faxed. The form is immediately reported to the bidding unit, the leader unit, the ethics committee of the research unit, the State Food and Drug Administration (CFDA), and the food and drug administration of the province (province or city) in the area where the researcher is located.

Serious adverse events should be documented in detail, symptoms, time of onset, time of treatment, measures taken, time and manner of follow-up, and outcomes. If the investigator believes that a serious adverse event is not related to the modified technique and is potentially related to the study condition, then this relationship should be described on the Serious Adverse Events page of the Medical Record Report Form. If the strength of a serious adverse event that is occurring or its relationship to the modified technique changes, a follow-up report of the serious adverse event should be sent immediately to the sponsor. All serious adverse events should be followed up to recovery or stabilization.

7. Data Management

7.1 Statistical analysis datasets

Full Analysis Set: pain score, operation time, efficacy analysis was performed on all

randomized cases.

Per-protocol Set: All cases that meet the trial protocol. No imputation is made for missing data. The parameters was simultaneously analyzed statistically for datas.

Safety Analysis Set: All patients enrolled in the study were included in the safety analysis set. This data set is used for security analysis.

8. Quality Assurance

Researchers must be clinically trained physicians and work under the direction of a senior professional.

Pre-test clinical wards must meet the requirements of standardization to ensure that the rescue equipment is fully equipped.

The research center must be carried out in strict accordance with the research plan and truthfully entered the case report form.

The auditor should follow the standard operating procedures, supervise the clinical trials, confirm that all data records and reports are correct and complete, all case report forms are entered correctly, and consistent with the original data to ensure that the trials are carried out in accordance with the clinical research protocol.

In the event of SAE, each research unit shall be notified in a timely manner and the study shall be temporarily suspended if necessary.

The research units participating in the trial should accept the audits of the sponsors and the drug regulatory authorities. It is especially important that the researchers and their related personnel should provide convenience and time for the inspection and audit.

9. Dissemination of Results and Publication Policy

9.1 Use of Information

The Scientific Committee has full access to the final data so that appropriate academic analysis and reporting can be conducted on the results of the study.

9.2 Publish

All investigators and committee members fully authorize the Scientific Committee to publish the results for the first time or for the first time. No other publications are allowed until the first publication. Any subsequent publication or publication of the study participants (including sub-studies) must be approved by the Scientific Committee and cited for the study and initial publication.

The final decision of any manuscript/summary/newsletter is made by the Scientific Committee after the company has been notified in advance (for internal review and comments). The sponsor may request the name of the sponsor and/or the name of one or more of the sponsors listed or not listed in this publication.

The sponsor may delay the publication or communication for a limited period of time in order to protect the confidentiality or ownership of any information contained therein.

10. Duration of the Project

Start time: June 2016, and planned end time: March 2018.

11. Project Management

11.1 Sponsor Obligations

The Sponsor monitor or designee will contact and visit the Investigator regularly and will be allowed, on request, to inspect the various records of the trial. The monitor will visit as soon as possible following enrollment of the first subject and at regular intervals during the study as deemed necessary. It will be the monitor's responsibility to inspect the source documents at regular intervals throughout the study, to verify the adherence to the protocol and the completeness, correctness and accuracy of all eCRF entries. The study monitor will have access to laboratory test reports and any other source records and data needed to verify the entries on the eCRFs, unless restricted by local laws. The Investigator agrees to cooperate with the study monitor to ensure that any problems detected in the course of these monitoring visits are resolved.

This study will be conducted in compliance with Good Clinical Practice, and applicable regulatory requirements.

11.2 Investigator Obligations

The investigator is responsible for ensuring that the study is performed in accordance with the protocol, current ICH guidelines on Good Clinical Practice (GCP), and applicable regulatory and country-specific requirements.

12. Ethics

12.1 Ethical Principles

The study will be conducted in accordance with the principles established by the 18th World Medical Association Joint Conference (Helsinki, 1964) and all subsequent amendments.

12.2 Laws and regulations

This study will be conducted in accordance with all laws and regulations.

12.3 Data Protection

Patient personal data and researcher personal data that may be included in the database shall be handled in accordance with all applicable local laws and regulations.

When archiving or processing personal data relating to the investigator and/or patient, shall take all appropriate measures to protect and prevent any unauthorized third party from obtaining such information.

12.4 Confidentiality of Subject Records

The researcher should keep all information received, obtained or derived during the course of the study confidential and take all necessary steps to ensure that it is not compromised.

12.5 Record Retention

The investigator should arrange for the storage of the research documents until the end of the study. In addition, for patient record keeping, researchers should follow specific local regulations/guidance.

Unless otherwise stated in the investigator's agreement, in accordance with other standards and/or local laws, the investigator is advised to keep the research document for at least five years after the study is completed or interrupted.

12.6 Study early interruption

If the researcher decides to withdraw from the study, he/she must notify the study team in writing.

If applicable, the Ethics Committee (IRB) and the health supervisory authority should be notified in accordance with local regulations.

12.7 Audit and Inspection

The investigator agrees to have the sponsor's inspectors/regulators' inspectors directly review the subject's research records for review and understand that these individuals are bound by the principle of occupational confidentiality and therefore should not disclose any personal identity or personal medical care of the patient’s information.

The investigator will make every effort to assist in the conduct of inspections and inspections, so that the inspectors/inspectors have access to all necessary equipment, data, and documentation.

The confidentiality of the verification data and the protection of the patient should be

respected during these inspections.

The researcher should immediately communicate the results and information given by the regulatory agency to the sponsor.

The investigator shall take appropriate measures at the request of the sponsor to take corrective action on all issues identified during the inspection or inspection process.

13. Informed Consent Forms

Prior to participation, the study procedures and any known or likely risks will be explained to the subjects by the investigator or other medically qualified co-investigator. An Informed Consent Form will also be provided containing all the required information. Any questions will be answered and the patient will then be given sufficient time to consider their participation in the study before signing a consent form. Subjects should receive a copy of the Informed Consent Form.

The Investigator will explain that the subjects are completely free to refuse to enter the study or to withdraw from it at any time, without any consequences for their further care and without the need to justify.

Each subject will be informed that the subject's source medical records may be checked by representatives from the Sponsor or from a regulatory agency, in accordance with applicable regulations. However, they should be made aware that all information will be treated with confidentiality, and a study ID code or number will identify them.

14. Financing and Insurance

14.1 Financial Disclosure

The investigator is responsible for updating the Sponsor if there are any changes that would affect their Financial Disclosure during the conduct of the study.

14.2 Liability and Insurance Conditions

In case of any damage or injury occurring to a subject in association with the participation in the study, Dianjiang County People's Hospital of Chongqing has insurance covers.
